# Altered Intrinsic and Casual Functional Connectivities of the Middle Temporal Visual Motion Area Subregions in Chess Experts

**DOI:** 10.3389/fnins.2020.605986

**Published:** 2020-12-01

**Authors:** Limei Song, Yanming Ge, Jinfeng Long, Peng Dong

**Affiliations:** ^1^School of Medical Imaging, Weifang Medical University, Weifang, China; ^2^Medical Imaging Center, Affiliated Hospital of Weifang Medical University, Weifang, China

**Keywords:** visuospatial attention network, resting-state functional connectivity, chess experts, visual motion area, granger causality analysis

## Abstract

An outstanding chess player needs to accumulate massive visual and spatial information for chess configurations. Visual motion area (MT) is considered as a brain region specialized for visual motion perception and visuospatial attention processing. However, how long-term chess training shapes the functional connectivity patterns of MT, especially its functional subregions, has rarely been investigated. In our study, using resting-state functional connectivity (RSFC) and Granger causality analysis (GCA), we studied the changed functional couplings of MT subregions between 28 chess master players and 27 gender- and age-matched healthy novices to reveal the neural basis of long-term professional chess training. RSFC analysis identified decreased functional connections between right dorsal-anterior subregion (CI1.R) and left angular gyrus, and increased functional connections between right ventral-anterior MT subregion (CI2.R) and right superior temporal gyrus in chess experts. Moreover, GCA analyses further found increased mutual interactions of left angular gyrus and CI1.R in chess experts compared to novice players. These findings demonstrate that long-term professional chess training could enhance spatial perception and reconfiguration and semantic processing efficiency for superior performance.

## Introduction

Chess playing is a complex intellectual game that is considered as a hard mental activity that requires sophisticated problem-solving skills. During chess playing, many kinds of cognitive processes are involved, e.g., working memory, attention, visuospatial perception, motivation, and decision making (Onofrj et al., 1995; Amidzic et al., 2001; van der Maas and Wagenmakers, 2005). The daily skill practice can result in the representative neural structural and functional changes that underlie the related particular skill (Maguire et al., 2000; Gaser and Schlaug, 2003a,b; Draganski et al., 2004; Zou et al., 2012; Song et al., 2020). Chess is seen as a typical example for an expertise task requiring domain-specific experience to study brain structural and functional plasticity. Therefore, to reveal the functional changes, master chess players provide a good opportunity to uncover the neural basis of brain plasticity induced by long-term skill practice (Zhou et al., 2018).

Chess players acquire outer information such as chess objects and position information on the board mainly relying on visual inputs. To become an outstanding chess player, one needs to accumulate massive visual experience of chess configurations. This experience confers distinct advantages in chess experts when encountering situations that commonly appear in games compared with novices. Increasing evidence has demonstrated that chess expertise can result in changed interactions between different brain networks due to frequent practice (Bilalic et al., 2011; Krawczyk et al., 2011; Duan et al., 2012a,b, 2014; Song et al., 2020), especially visuospatial attention network, which includes dorsal and ventral attention networks playing a key role in preparing, applying goal-directed selection for stimuli and responses, and detecting the salient targets (Corbetta and Shulman, 2002; Astafiev et al., 2004; Kincade et al., 2005; Fox et al., 2006; Wang et al., 2016; Wu et al., 2016). Visual motion area (MT), which is traditionally considered to be specialized for the perception of motion in the visual modality, plays an important role in visual motion and visuospatial attention processing and is the hub of the visuospatial attention network (Gao et al., 2020). Recently, based on meta-analysis in BrainMap database and task-related coactivation-based parcellation approach, Gao et al. (2020) identified the MT region and parcellate this area into several different functional subregions showing different coactivation patterns and functions. Revealing the specific changes of MT subregions with a fine-grained MT atlas in master chess players compared to novices could provide a new avenue to delineate the neural basis of long-term skill practice on brain plasticity.

In this study, we studied the specific changes of functional connectivity patterns of each MT subregion in chess experts to uncover the neuroanatomical basis of brain plasticity induced by long-term chess practice using a full data-driven approach. First, bilateral MT subregions were defined with a fine-grained MT atlas (Gao et al., 2020), and the whole-brain resting-state functional connectivity (RSFC) analysis for each subregion was performed. Next, Granger causality analysis (GCA) was used to identify the causal interactions between MT subregions and regions showing changed RSFC. Finally, correlation analyses were done to reveal the associations between changed RSFC or GCA and behavioral performances.

## Materials and Methods

### Participants

Twenty-eight chess players (female/male = 10/18, mean ± standard deviation of age = 27.64 ± 9.15 years; mean ± standard deviation of education = 13.43 ± 2.71 years) and 27 novice players (female/male = 15/12, mean ± standard deviation of age = 26.37 ± 6.68 years; mean ± standard deviation of education = 14.24 ± 3.06 years) were used in this study. The data were accessed from the “1000 Functional Connectomes Project”^1^. The professional chess players had been seriously training regularly (training time: 4.17 ± 1.72 h/day), whereas the 27 gender-, age-, and education-matched (gender: *p* = 0.14; age: *p* = 0.57; education: *p* = 0.31) novice players understand the rules and simple strategies only for playing Chinese chess. All the sample characteristics are listed in Table 1. No differences on observation skills or clear-thinking ability were found between these two groups (Li et al., 2015). All participants were right-handed and had no history of psychiatric or neurological disorders. Written informed consent was obtained from each subject, and approval was obtained through the local institutional review board of the West China Hospital of Sichuan University. The detailed information of this dataset can be found in Li and colleagues’ study (Li et al., 2015).

**TABLE 1:** Demographics of chess and novice players used in the present study.

	Chess players (*n* = 28)	Novice players (*n* = 27)	*p* value
Gender (male/female)	18/10	12/15	0.14
Age (years) (mean ± SD)	27.64 ± 9.15	26.37 ± 6.68	0.57
Education (years) (mean ± SD)	13.43 ± 2.71	14.24 ± 3.06	0.31
FD_power (mean ± SD)	0.26 ± 0.25	0.21 ± 0.07	0.33

*A Pearson χ^2^ test was used for gender comparison. Two-sample *t* tests were used for age, education, and FD values comparisons. FD, frame displacement.*

### Resting-State Functional Magnetic Resonance Imaging Data Acquisition

The resting-state functional magnetic resonance imaging (fMRI) data were acquired for each subject using a Siemens 3.0-T MRI scanner at the MR Research Center of West China Hospital of Sichuan University, Chengdu, China. All MR procedures were performed when subjects were relaxed with their eyes open and fixated on a cross-hair centered on the screen. A T2-weighted gradient-echo echo-planar imaging (EPI) sequence was used to collect the resting-state fMRI scans. A total of 205 whole-brain echo-planar pulse sequence volumes were acquired using the following parameters: repetition time = 2,000 ms; echo time = 30 ms; flip angle = 90°; axial slice thickness = 5 mm, with no gap; slice number = 30; and voxel size = 3.75 × 3.75 × 5 mm^3^.

### Resting-State fMRI Data Preprocessing

The resting-state fMRI scans were preprocessed including the following steps. The first 10 volumes were discarded to facilitate magnetization equilibrium, and the remaining images were realigned to the first volume to correct head motion. Next, all fMRI scans were normalized to the Montreal Neurological Institute EPI template and resampled to 3 × 3 × 3 mm^3^. The fMRI scans were smoothed using a Gaussian kernel of 6-mm full width at half maximum. Friston-24 head motion parameters, white matter, cerebrospinal fluid, and global mean signals were then regressed out. Finally, the functional images were filtered with a temporal band path of 0.01 to 0.1 Hz. To further exclude the head motion effects on functional connectivity analysis, a scrubbing method was conducted to censor each subject’s bad fMRI scans to find out the mean frame displacement, which was above 0.5 mm, and one volume before and two volumes after the bad volume were discarded (Power et al., 2012). Only the participants with the fMRI scans more than half of the total time points were kept for the following analyses. There is no significant difference in head motion between these two groups (*p* = 0.33; Table 1).

### Definition of the MT Subregions

**FIGURE 1:**
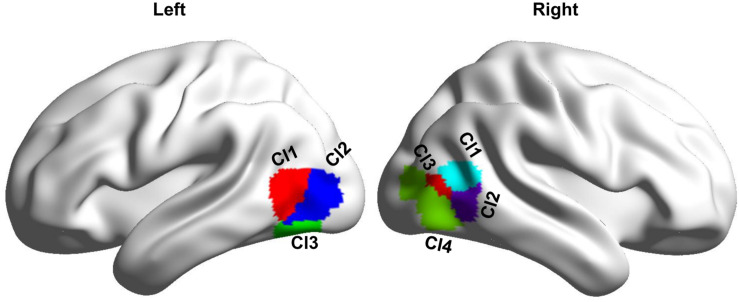
Definition of the bilateral MT subregions based on a recent MT atlas created by coactivation-based parcellation. **Left** MT is parcellated into three subregions, and **right** MT is parcellated into four subregions.

MT subregions were defined with an MT atlas which, was created using coactivation-based parcellation approach (Gao et al., 2020). In this atlas, the left MT was parcellated into three subregions including two dorsal subregions (Cl1 and Cl2) and one ventral subregion (Cl3), and the right MT was parcellated into four subregions including two anterior subregions (Cl1 and Cl2), one middle subregion (Cl3), and an additional posterior subregion (Cl4) (Gao et al., 2020; Figure 1).

### Functional Connectivity Analyses

To explore whether the whole-brain functional connectivity showed differences in the MT subregions between chess and novice players, a whole-brain functional connectivity map defined by the Pearson correlation of each MT subregion for each subject was calculated. To improve normality, Fisher’s *z* transformation was applied to change the *r* values to *z* values. To identify the functional connectivity pattern differences between chess experts and novices, two-sample *t* tests were performed in a voxel-wise manner, and the significant level was determined with a cluster-level Monte Carlo simulation–corrected threshold of *p* < 0.05 (cluster-forming threshold at voxel-level *p* < 0.001).

### GCA for Chess and Novice Players

Granger causality analysis was performed to further explore the causal interaction of brain areas with changed RSFC between chess and novice players. The mean time series of these brain areas were extracted, and the bivariate GCA was performed on the time series to test the causal interactions. The order of the autoregressive model used for computation of the influence measure was selected using the Bayesian information criterion, and magnitudes of causal interactions were obtained for each subject (Wang et al., 2017a, 2019). GCA was performed using the Causal Connectivity Toolbox (Seth, 2010). Two-sample *t* tests were performed on the magnitudes of causal interactions to identify the significant causal influences between brain areas using a significant threshold of *p* < 0.05 corrected by Bonferroni correction.

### Correlation Analysis

To explore the relationship between changed resting-state functional or causal connectivities and the amount of time that chess players spent on professional training, correlation analyses were performed, and the significant level was set at *p* < 0.05.

## Results

### Differences in Functional Connectivities of MT Subregions

**FIGURE 2:**
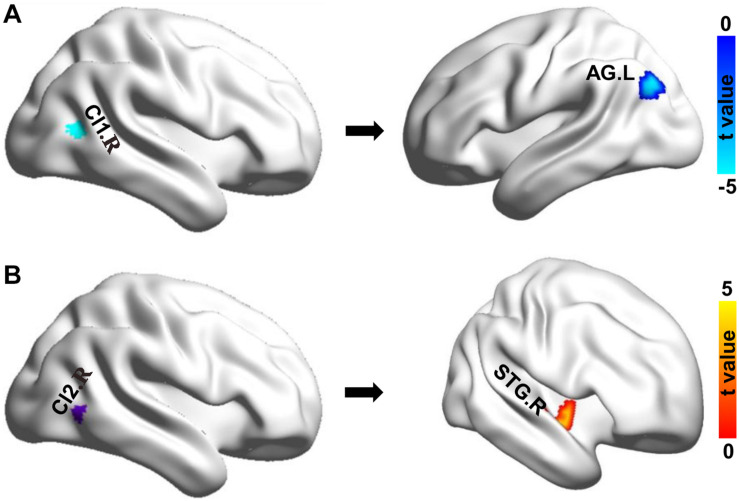
Changes in resting-state functional connectivity. **(A)** Chess experts showed decreased functional connectivity between Cl1.R and left angular gyrus (AG.L). **(B)** Chess experts showed increased functional connectivity between Cl2.R and right superior temporal gyrus (STG.R) compared with novice players.

Whole-brain RSFC analyses identified only significant differences of right MT subregions in chess players. The right Cl1 (CI1.R) showed significantly reduced functional connectivity with left angular gyrus (AG.L) in chess players compared with novices (Figure 2). A significantly increased functional connectivity between right Cl2 (CI2.R) and right superior temporal gyrus (STG.R) was found in chess experts (Figure 2).

### Differences in Casual Interactions

**FIGURE 3:**
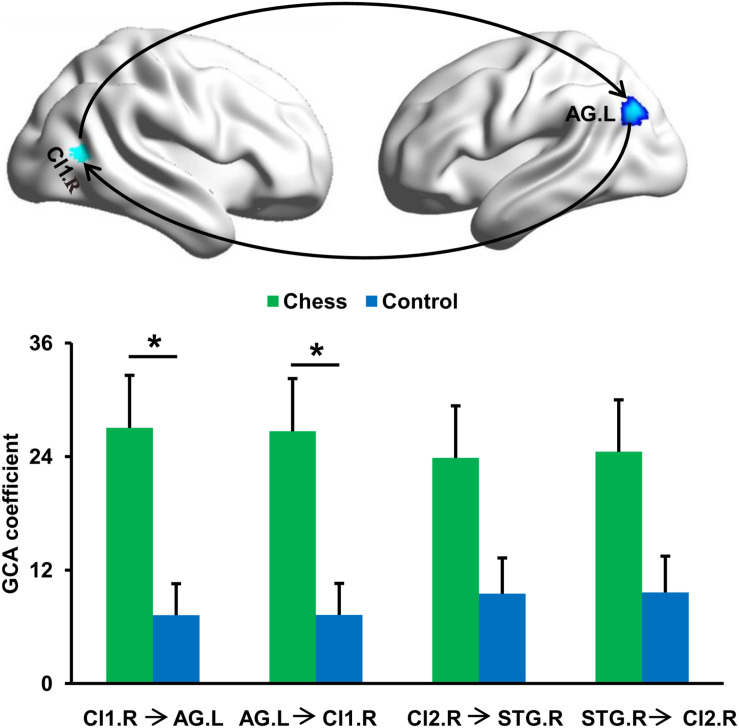
Changes in effective connectivities. Chess experts showed increased mutual effective connectivity between Cl1.R and AG.L compared with novice players. No significant difference in effective connectivity between Cl2.R and STG.R in chess experts was found compared with novice players. ‘*’ represents the significant difference.

To further determine the differences in causal interactions, the GCA was performed and identified a significantly increased magnitude of mutual causal interactions between Cl1.R and AG.L in chess players compared with novices (Figure 3). Chess players showed no significant differences in magnitude of causal interactions between Cl2.R and STG.R (Figure 3).

### Correlation Analyses

Correlation analyses did not find significant associations between the functional and effective connectivity strength and the amount of time chess players spending on professional training (all *p* > 0.05).

## Discussion

In the present study, using resting-state functional MRI, we revealed that specifically long-term chess training can change the functional interactions of MT subregions. Connectivity analyses identified significantly increased RSFC of Cl1.R and Cl2.R in chess experts compared with novices. Specifically, decreased functional connectivity between Cl1.R and AG.L and increased functional connectivity between Cl2.R and STG.R were found in chess experts. In addition, GCA analysis further revealed chess experts showed enhanced mutual interactions between Cl1.R and AG.L. These findings suggest chess visual practice may enhance the neuroplasticity of Cl1.R and Cl2.R in MT, and the increased functional connections resulting from the professional chess training may enhance visuospatial attention and semantic and auditory memory processing.

MT is approximately located in the junction of the posterior middle temporal gyrus and occipital gyrus (Smith et al., 2006). Increasing evidence demonstrates that MT is a functional complex brain area with several distinct functional subregions (Smith et al., 2006; Kolster et al., 2010). Recently, using meta-analysis of visual motion task and coactivation-based parcellation approach with BrainMap database, Gao and colleagues identified the bilateral MT in human and subdivided this area into several different functional subregions (Gao et al., 2020). By comparing the whole-brain functional connectivity maps, we demonstrate that long-term professional chess training could induce the functional plasticity of different subregions. Moreover, neurons in MT are highly sensitive to the speed and direction of visual stimuli in motion (Albright, 1984). Previous studies have revealed that daily chess practice could activate this area during chess-related visual search tasks for processing visually presented chess game positions (Bilalic et al., 2010, 2011) and could change the RSFC in a behaviorally specific manner (Hampson et al., 2006; Lewis et al., 2009). In our study, we found that long-term chess practice can result in significantly changed RSFC patterns of MT in chess experts. The changed functional connections in MT subregions suggest Cl1.R and Cl2.R are the critical hub regions for integrating visual information processing in chess experts.

Chess experts showed enhanced functional connectivity between Cl2.R and STG.R. STG is an association cortex and plays a key role in auditory memory (Rajarethinam et al., 2000; Zevin, 2009; Wang et al., 2015). Auditory memory involves auditory reception of orally presented information, auditory processing and storing, and finally recalling what has been heard. A previous study reported that auditory memory pathways involved during the chess game can be affected by the process of long-term chess playing (Fattahi et al., 2015). Fattahi et al. (2015) found that chess experts had better auditory memory function than non–chess players. Besides, several studies have demonstrated MT region is also related to motion of auditory (Poirier et al., 2006; Watkins et al., 2013). The increased functional couplings between Cl2.R and STG.R suggest long-term chess practice could strength auditory memory to advance better performances during the chess game. In addition, several studies showed that STG is implicated in the processing of eye movements, as well as in the visual analysis of social information conveyed by gaze and body movement (Allison et al., 2000; Hoffman and Haxby, 2000; Pelphrey et al., 2005). The long-term and intensive visual practice of chess expertise may elevate functional interactions between Cl2.R and STG.R in chess experts for excellent concentration in devoting to the game.

We also found decreased functional connectivity but increased mutual effective connectivities between Cl1.R and AG.L in chess players. AG.L is primarily involved in reading and semantic processing (Wang et al., 2017b). Previous studies about reading also revealed that AG.L is involved in orthography to semantic mapping (Horwitz et al., 1998). Thus, the decreased functional connectivity between AG.L and Cl1.R may be related to higher efficiency and less effort for semantic processing related to chess game. This conclusion is supported by the increased mutual effective connectivities between these two areas. The increased effective connectivities between Cl1.R and AG.L indicate faster button-up and top-down information communication in chess experts than in novices.

There are several limitations to the present study. First, the number of the included chess players is small. A larger sample is needed to further validate the current findings. Second, our study focused only on resting-state functional connections. A topological approach of structural brain connectivity networks should be explored in future studies to reveal the anatomical basis of any functional changes. Third, as the technical limitation of fMRI acquisition, the GCA of fMRI data is still controversial. Our findings obtained using GCA still need to be validated with other techniques.

## Conclusion

Using resting-state functional and GCAs, we revealed decreased functional connectivity between CI1.R and AG.L, increased functional connectivity between CI2.R and STG.R, and increased mutual effective connectivities between Cl1.R and AG.L in chess experts. These results indicated long-term professional training could modulate the functions of MT area. Our findings also suggested that CI1.R and CI2.R have a critical effect on chess visual practice, and increased functional connections between CI2.R and STG.R may in turn facilitate communication within the network.

## Data Availability Statement

Publicly available datasets were analyzed in this study. This data can be found here: http://fcon_1000.projects.nitrc.org/indi/pro/wchsu_li_index.html.

## Ethics Statement

The studies involving human participants were reviewed and approved by the local Institutional Review Board of the West China Hospital of Sichuan University. The patients/participants provided their written informed consent to participate in this study.

## Author Contributions

LS and PD designed the study. LS, YG, JL, and PD performed the research. LS and JL analyzed the data. LS, YG, and PD wrote the manuscript. All authors contributed to the article and approved the submitted version.

## Conflict of Interest

The authors declare that the research was conducted in the absence of any commercial or financial relationships that could be construed as a potential conflict of interest.

## Footnotes

1 http://fcon_1000.projects.nitrc.org/indi/pro/wchsu_li_index.html
